# Exploring the causal association between uric acid and lung cancer in east Asian and European populations: a mendelian randomization study

**DOI:** 10.1186/s12885-024-12576-0

**Published:** 2024-07-05

**Authors:** Ping Lin, Linxiang Zhang, Xiaohui Tang, Jihuang Wang

**Affiliations:** 1 Department of Radiotherapy, The Second Hospital of Longyan, Longyan, 364000 Fujian Province China; 2 Department of Dermatology, The Second Hospital of Longyan, Longyan, 364000 Fujian Province China; 3 Department of Pathology, The Second Hospital of Longyan, Longyan, 364000 Fujian Province China

**Keywords:** Uric acid, Lung cancer, Protective factor, Mendelian randomization

## Abstract

**Background:**

Lung cancer still ranks first in the mortality rate of cancer. Uric acid is a product of purine metabolism in humans. Its presence in the serum is controversial; some say that its high levels have a protective effect against tumors, others say the opposite, that is, high levels increase the risk of cancer. Therefore, the aim of this study was to investigate the potential causal association between serum uric acid levels and lung cancer.

**Methods:**

Mendelian randomization was used to achieve our aim. Sensitivity analyses was performed to validate the reliability of the results, followed by reverse Mendelian analyses to determine a potential reverse causal association.

**Results:**

A significant causal association was found between serum uric acid levels and lung cancer in East Asian and European populations. Further sublayer analysis revealed a significant causal association between uric acid and small cell lung cancer, while no potential association was observed between uric acid and non-small cell lung cancer, squamous lung cancer, and lung adenocarcinoma. The sensitivity analyses confirmed the reliability of the results. Reverse Mendelian analysis showed no reverse causal association between uric acid and lung cancer.

**Conclusions:**

The results of this study suggested that serum uric acid levels were negatively associated with lung cancer, with uric acid being a potential protective factor for lung cancer. In addition, uric acid level monitoring was simple and inexpensive. Therefore, it might be used as a biomarker for lung cancer, promoting its wide use clinical practice.

## Introduction

Lung cancer (LC) remains one of the most common malignant tumors worldwide, with the highest mortality rate. According to the 2023 Cancer Statistics, LC is the second most common malignant tumor, while the mortality rate is the highest for cancer [1].In the past decades, the survival rate of LC has been improved to some extent as a result of the popularization of early screening, the widespread use of low-dose computed tomography, and the development of targeted therapy and immunotherapy, but its 5-year survival rate is also only 19% [2], mainly due to the fact that more than half of the patients have a locally advanced tumor or distant metastasis at the time of diagnosis [3].Therefore, LC is a serious danger to human health.

The relationship between metabolic changes and cancer has attracted much attention in recent years [4]. Uric acid (UA)is one of the purine metabolism of the body working as a powerful antioxidant [5]. Indeed, it modulates superoxide dismutase activity, scavenges free radicals, and chelates transition metals, thereby protecting cells from oxidative damage [6]. Purine bases are converted to UA by xanthine oxidoreductase (XOR), which produces other antioxidants that from the innate immune system [7].XOR plays an anticancer or carcinogenic role depending on the types of tumors [8]. The role of UA in tumors is still controversial; Horsfall et al. found that high UA levels have a protective effect on tumor cells [9], while Wang et al. found the opposite, that is, high UA levels increase the risk of cancer [10]. Thus, the causal relationship between serum UA levels and cancer remains currently unclear. Mendelian randomization (MR) is a relatively effective analytical method that uses genetic variation as instrumental variables (IVs) to discover the potential causal associations between exposures and outcomes. The IV model reduces confounding factors [11]. LC is categorized into two main groups according to the pathological types: small cell lung cancer (SCLC) and non-small cell lung cancer (NSCLC), the latter including lung squamous cell carcinoma (LUSC) and lung adenocarcinoma (LUAD) [12].Thus, in this work, a two-sample MR method was used to investigate the causal association between serum UA levels and LC and its subtypes.

## Materials and methods

### Study design

**Fig. 1:**
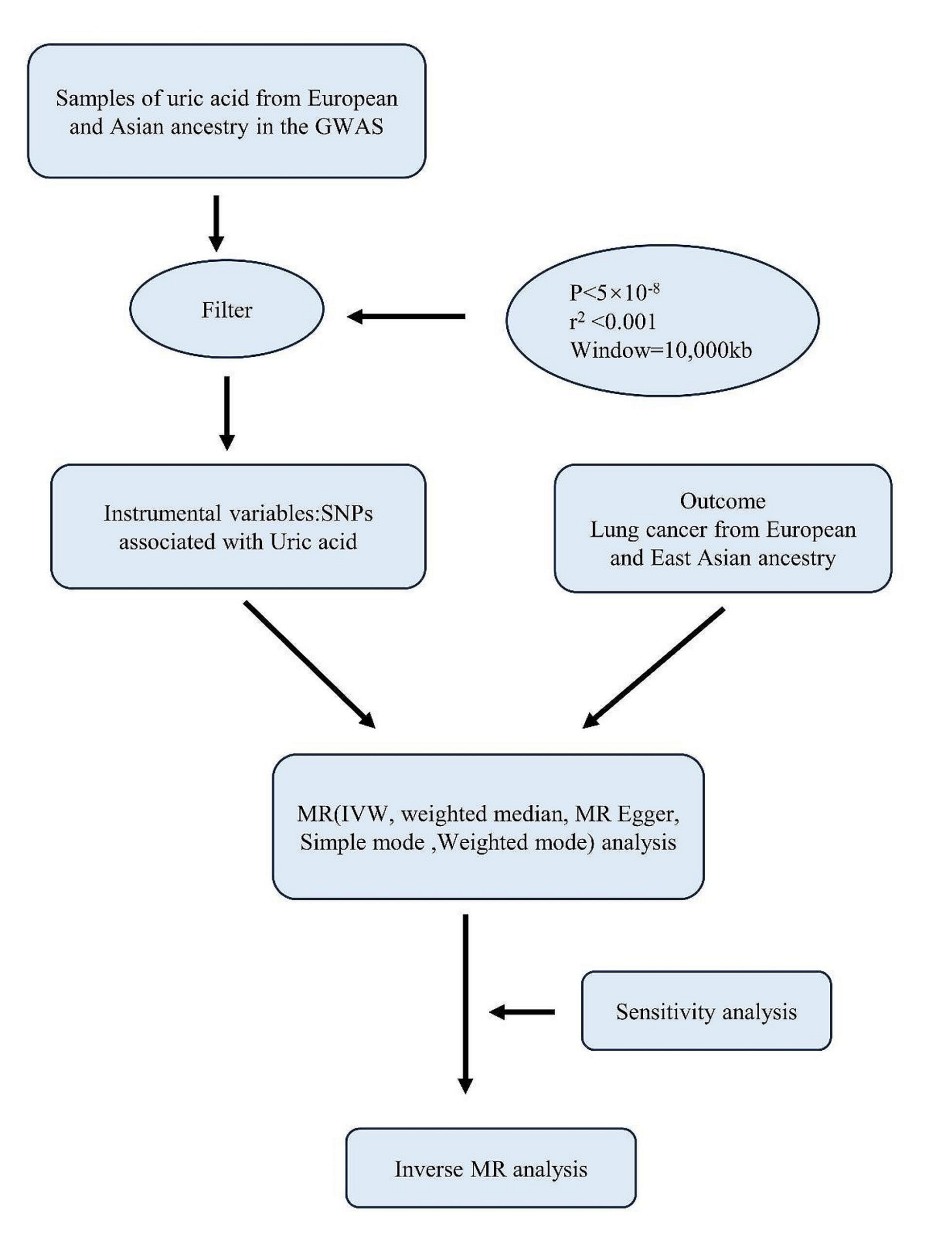
Study design

A two-sample MR method was used to investigate the causal associations between serum UA levels and LC and its subtypes in East Asian population and European population. Firstly, single nucleotide polymorphisms (SNPs) associated with serum UA and LC (LUSC, LUAD, NSCLC, SCLC) in East Asian and European populations were used as IVs and obtained from the Genome-Wide Association Studies (GWAS) database (https://gwas.mrcieu.ac.uk/). Next, the inverse variance weighted (IVW), weighted median, MR Egger, and weighted mode were used to evaluate the causal association between serum UA and LC. A schematic design of the study is shown in Fig. 1.

### Data sources

Study data were obtained from the GWAS database. The data of the exposure variable UA were derived from the bbj-a-57 dataset of the East Asian population, and included 109,029 samples and 6,108,953 SNPs. The data of the exposure variable UA was derived from the ebi-a-GCST90018977 dataset of the European population and included 343,836 samples and 19,041,286 SNPs. The summary data of the outcome variable LC in this study were derived from the bbj-a-133 dataset and included 4,050 cases, 208,403 controls, and a total of 8,885,805 SNPs. The European population was derived from the ebi-a-GCST90018875 dataset and included 3,791 cases, 489,012 controls, and a total 492,803 SNPs (Table 1). The potential causal associations between UA and different pathological types of LC were further investigated using LC subtype-specific data for subgroup analysis due to the heterogeneity of different pathological types. All data were obtained from the GWAS database, thus, no ethical approval was required for this study.

**Table 1:** Basic information of the study population

Phenotype	GWAS ID	Population	Case(*n*)	Controls(*n*)	Number of SNPs
Exposure					
UA	bbj-a-57	East Asian	109,029	/	6,108,953
UA	ebi-a-GCST90018977	European	343,836	/	19,041,286
Outcome					
LC	bbj-a-133	East Asian	4,050	208,403	8,885,805
LC	ebi-a-GCST90018875	European	3,791	489,012	492,803
SCLC	finn-b-C3_SCLC	European	179	218,613	16,380,466
NSCLC	finn-b-C3_LUNG_ NONSMALL _EXALLC	European	1,627	174,006	16,380,305
LUSC	finn-b-C3_NSCLC_SQUAM	European	365	218,427	16,380,466
LUAD	finn-b-C3_NSCLC_ADENO	European	571	218,221	16,380,466

### Statistical analysis

MR analyses relied on three plausible assumptions:①UA as IVs is strongly associated with exposure (association).②UA as IVs is not associated with confounders (independence).③UA as IVs affects outcomes only through exposure (exclusion restriction criteria). In this study, both exposure-related IVs and outcome-related IVs were from two independent samples of the same ethnicity. A rigorous approach was used to select exposure strongly associated to SNPs as IVs using the GWAS pooled data described above. Screening conditions were defined as statistically significant genome-wide at *P* < 5 × 10^− 8^. The linkage disequilibrium was set to r^2^ < 0.001 within a clumping window of 10,000 kb to prevent biased results due to it. Finally, the statistic considering F > 10 unlikely affected by the bias of weak IVs was computed to address the issue of bias due to weak IVs. F was calculated as follows: F = Beta^2^/SE^2^, where Beta and SE represent the correlation and standard deviation of genes and exposure, respectively. The SNPs filtered according to the above conditions were matched with the LC dataset.

### Methods of causality verification

IVW was mainly used to evaluate the causal association between serum UA levels and LC, while weighted median method, MR Egger, and weighted model methods were performed to validate it. The sensitivity analysis of the results was also performed to explore potential heterogeneity and pleiotropy. The heterogeneity of the MR results was assessed using the Cochran’s Q test. The random-effects model of IVW was used in case of heterogeneity. Multiple validity was assessed using the MR-Egger intercept analysis. Finally, the “leave-one-out” analysis was used to perform the sensitivity analysis. All data in this study are available online, and analyzed using R software (R4.3.2) and the “TwoSampleMR” package. A value of *P* < 0.05 was considered statistically significant.

### Inverse MR analysis

Reverse MR analysis was performed using LC SNPs as IVs and UA as the outcome IVs investigate whether LC has an effect on UA and whether the previously identified relationships were bidirectional.

## Results

### Selection of IVs

A total of 43 SNPs with genome-wide significance were obtained for UA in East Asian populations as well as 268 SNPs with genome-wide significance for UA in European populations using the “TwoSampleMR” of the R package, with the smallest F-statistic value of 30 for IVs, indicating a strong correlation between IVs and exposure. Therefore, all SNPs were used to determine the potential impact of UA levels on LC risk.

### MR analysis

**Fig. 2:**
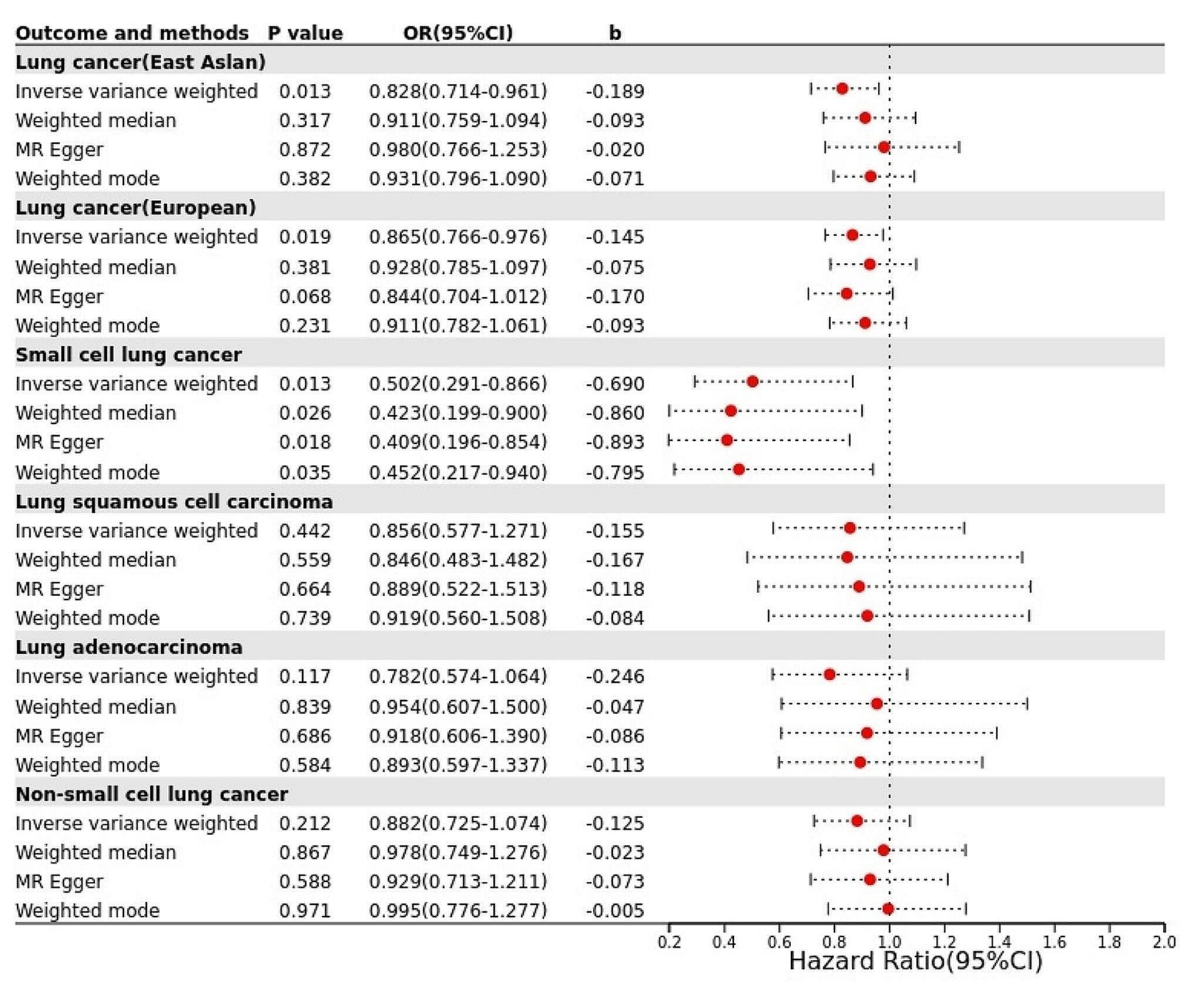
Forest plot of the results of two-sample MR analysis for UA and LC in East Asian and European populations

The potential correlation between UA and LC risk was analyzed by MR using serum UA levels as the exposure variable in East Asian and European populations and LC as the outcome variable. The results of the IVW method revealed that 42 SNPs revealed a statistically significant causal association between UA and LC in the East Asian population (OR = 0.828, 95% CI = 0.714–0.961, *P* = 0.013).While, 258 SNPs revealed a statistically significant causal association between UA and LC In the European population (OR = 0.865, 95% CI = 0.766–0.976, *P* = 0.019). The results of the IVW were considered decisive although the *P* values of the weighted median, the MR Egger and the weighted model were not statistically significant. The b-values were calculated in the same direction using all the four methods, with IVW as the most critical method, while the others were only used as a reference, and our results were considered statistically significant when the value of P of the IVW results was less than 0.05.Further analysis in the sub-stratum of LC showed no potential association of UA with NSCLC (*P* = 0.212), LUSC (*P* = 0.117), and LUAD (*P* = 0.442)for the time being, but the results of IVW showed a statistically significant causal association of UA with SCLC (OR = 0.502, 95% CI = 0.577-0.1.271, *P* = 0.013), The results of MR Egger, weighted median and weighted model were similar to the IVW results and statistically significant as well (*P* < 0.05), suggesting the preventive effect of UA on the development of LC (Fig. 2).

### Sensitivity analysis

**Fig. 3:**
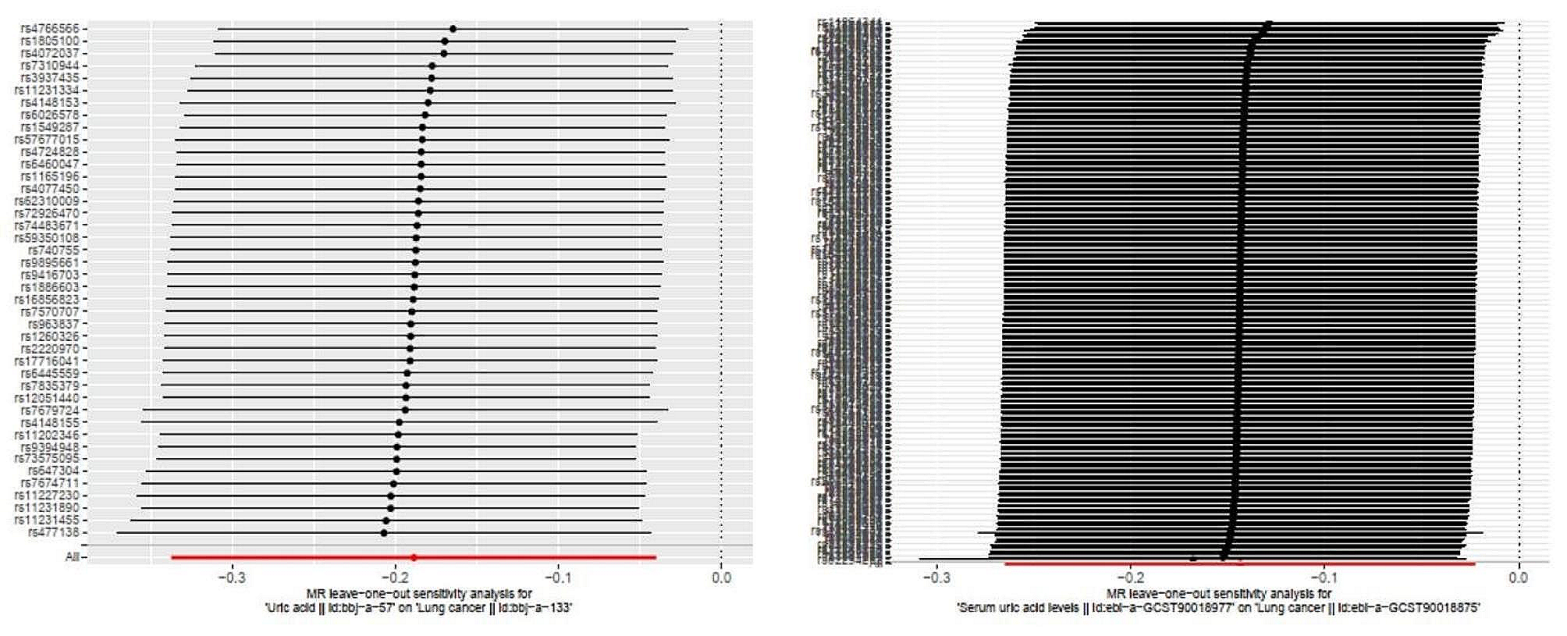
Sensitivity analysis of the leave-one-out method for UA and LC.

Heterogeneity analysis, horizontal pleiotropy analysis and leave-one-out analysis were performed to assess the reliability of the MR analysis results. Cochran’s Q test was used to assess the heterogeneity of the selected SNPs, resulting in no heterogeneity between UA and LC in the East Asian population, and between UA and SCLC in the European population (*P* > 0.05), However, a heterogeneous interference was found between UA and LC in the European population (*P* < 0. 05), and the results of our multiplicity test and leave-one-out analysis method were negative. Thus, the random-effects model of IVW was used, and the results showed that *P* = 0.019 calculated by the random-effects model of IVW, suggesting that these heterogeneities had a lesser impact on the results of our analysis. A causal association between UA and LC was found in the European population based on the random-effects model of IVW, which was our main method of analysis. None of them were significant in the MR-Egger intercept test, indicating that none of them were horizontally pleiotropic (Table 2). The effect of individual SNPs on the overall results was further analyzed using the “leave-one-out” analysis, revealing no significant effect on the results regardless of which SNP were excluded, indicating that the MR results in this study were stable. This suggests that the association between UA and LC in East Asian and European populations was not driven by a single SNP, but by all functional SNPs together(Fig. 3).

**Table 2:** Heterogeneity test and horizontal pleiotropy test

Exposure	Outcome	Heterogeneity test			Horizontal pleiotropy test		
		Method	Cochran’s Q	Q_pval	Egger_intercept	Se	pval
UA	LC(East Asian)	MR Egger	51.211	0.110			
UA	LC(East Asian)	IVW	54.747	0.074	-0.013	0.008	0.104
UA	LC(European)	MR Egger	381.786	< 0.001			
UA	LC(European)	IVW	382.014	< 0.001	0.001	0.002	0.696
UA	SCLC	MR Egger	238.355	0.642			
UA	SCLC	IVW	239.007	0.648	0.009	0.011	0.420
UA	LUSC	MR Egger	265.944	0.194			
UA	LUSC	IVW	266.039	0.206	-0.002	0.008	0.838
UA	LUAD	MR Egger	237.541	0.656			
UA	LUAD	IVW	238.825	0.651	-0.007	0.006	0.258
UA	NSCLC	MR Egger	281.350	0.066			
UA	NSCLC	IVW	281.721	0.070	-0.002	0.004	0.569

### Inverse MR analysis

**Fig. 4:**
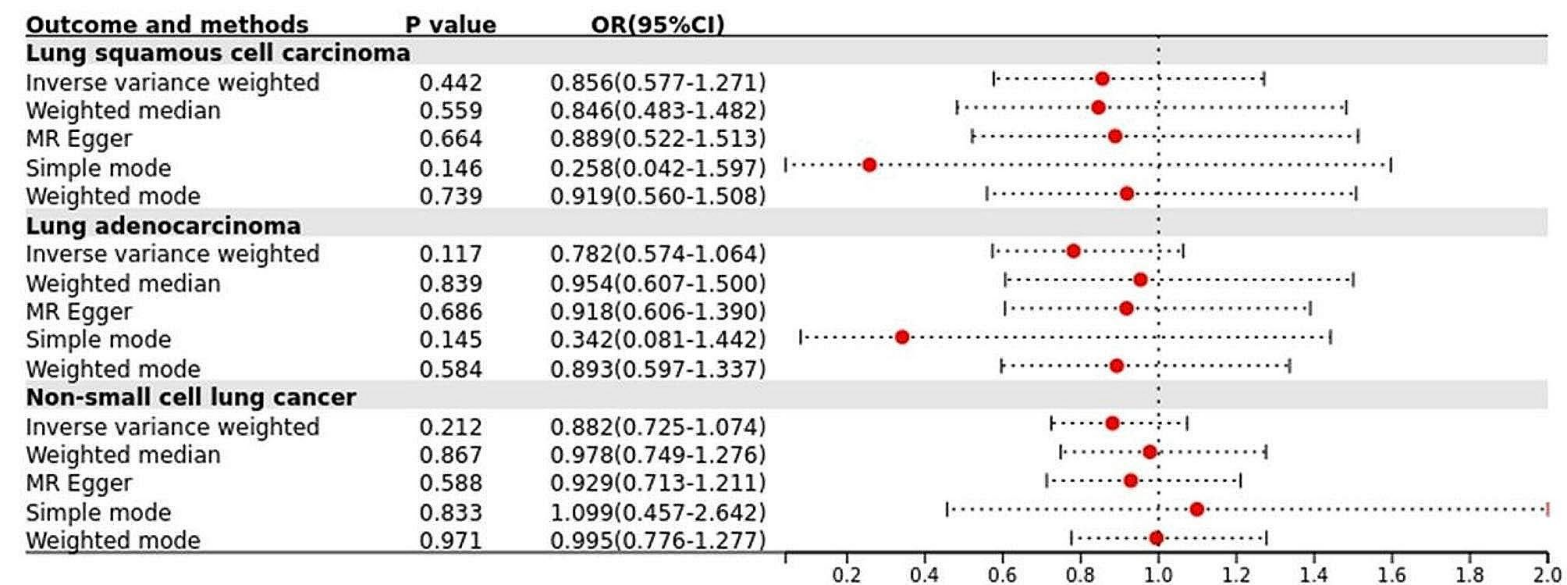
Forest plot of the results of inverse MR analysis for LC and UA in East Asian and European populations

A reverse MR analysis was performed to verify whether the observed UA was affected by LC, considering LC as the exposure variable and UA as the outcome variable. The results showed no evidence that LC affected UA in IVW (Fig. 4).

## Discussion

The prognosis of LC has improved thanks to the widespread use of low-dose computed tomography, the dissemination of early screening, and the development of targeted therapies and immunotherapy. However, it is still the leading cause of cancer death. Serum UA levels in the human body are mainly regulated by a balance among endogenous production, exogenous intake and excretion [13] Two-thirds of UA excretion passes through the kidneys and one-third through the intestine [14].UA is a double-edged sword in the human body, and its high levels lead to the development of several diseases, including gout, obesity, metabolic syndrome, diabetes, hypertension, chronic kidney disease [15].On the other hand, UA possesses antioxidant properties and neuroprotective effects, maintains blood pressure stability and regulates the body’s immunity, thus, low serum UA levels increases the risk of neurodegenerative diseases [16].Some studies showed that high UA levels activate the AKT/mTOR pathway, increase anabolic pathways, inhibit cellular autophagy, consequently promoting cell proliferation [17].In addition, sustained high UA levels trigger inflammation, as well as lipid cycling and storage, resulting in metabolic disorders and increased risk of cancer [18]. UA is a potent antioxidant, it is the most abundant free radical “scavenger” in human blood protecting cells from oxidative damage caused by free radicals, which increase cell mutation and promote tumor development, UA acts as a potent antioxidant and exerts anti-tumor development by reducing the occurrence of cellular mutation. Therefore, it has been hypothesized that high serum UA levels are associated with a reduced incidence of cancer and increased longevity in humans [5].Wang et al. demonstrated that UA pretreatment attenuates the cardiotoxicity of doxorubicin [19]. Yasutake et al. found that the antioxidant effect of UA ameliorates indomethacin-induced enteropathy in mice [20].The administration of UA at a certain dosage is safe and effective in improving the antioxidant ability of the body [21].Therefore, UA plays an important role in tumor development and treatment. However, the potential relationship between serum UA levels and LC risk is currently unknown.MR, is a new method of epidemiologic analysis, that reduces the effects of bias and confounding factors of traditional epidemiologic methods [22].The potential causal associations between serum UA levels and LC was assessed in this work using MR methods and the results revealed a significant causal associations between them in East Asian and European populations. Further sub-stratum analysis showed a significant causal association between UA and SCLC, but not between UA and NSCLC, LUSC, or LUAD, and the sensitivity analysis revealed that they were reliable results. The differences in the results of sublayer analysis might be related to the existence of heterogeneity of tumor cells in different pathological types. Tumor cells are reprogrammed by metabolism to perform their continuous proliferation, but tumor cells in different sites and different pathological types have different metabolic patterns and different metabolic reprogramming [23]; Thus, the differences in metabolic patterns might result in different results in different sublayer analyses. This might be also a potential reason for the causal association between UA and SCLC, and no causal association between UA and LUSC and LUAD. Therefore, serum UA level was negatively associated with LC risk. Reverse MR analysis also showed no reverse causal association between serum UA and LC.

XOR is involved in endogenous generation of UA. The reduction or deletion of XOR in tumor cells leads to decreased LC survival [24].Low XOR expression is associated with poor differentiation of breast, gastric, and colorectal cancers, increasing their risk of distant metastasis by more than two-fold [25].While, the inhibition or deletion of XOR increases the invasiveness of breast cancer cells [26]. Treatment of pancreatic cancer with gemcitabine induces the accumulation of intracellular UA, which mediates the expression of the major histocompatibility complex class I-associated chain A/B, thereby enhancing cellular sensitivity to killing [27]. XOR expression is enhanced in LC, and its metabolic breakdown produces UA that exerts a dual role in oxidative reactions, apoptosis, metabolism and immunity [8].The fact that UA is mainly excreted through the kidneys requires the involvement of multiple transporter proteins [28].Defects in urate transporter proteins contribute to several diseases including hypertension, and chronic kidney disease [29]. The substrates of certain transporter proteins are also protective against cancer, but transporter proteins affect drug and UA metabolism, also leading to cancer drug resistance and impaired UA metabolism [30]. Changes in SNPs at different loci affect the normal expression of genes [31]. SLC2A9 (rs7674711) is one of the solute transporter family members involved in blood UA metabolism, and the lack of this protein leads to renal hypouricemia [32]. ABCG2 protein (rs4148153, rs4148155) is one of the UA transporter proteins, and its increase in tumor cells leads to the resistance to multiple drugs. Hyperuricemia directly down-regulates ABCG2 expression [33]. High serum UA levels are the consequence of tumor cell injury. Urea and antigens released from apoptotic cells stimulate the immune system and the maturation of dendritic cells, thus inducing cytotoxic cell death, consequently inhibiting tumor cell proliferation and migration [34]. UA has a protective effect against LC, but the specific intrinsic mechanism is still unclear. Therefore, further clinical research is needed to discover it.

## Conclusions

Metabolic disorders are associated with the occurrence and development of tumors. The results of this study showed a significant negative correlation between UA level and LC risk, implying that UA might be a protective factor against LC development, as well as potentially ensuring a better prognosis to LC patients with high blood UA levels. In addition, the measurement of serum UA level is simple, inexpensive and easy to perform in clinical practice, suggesting that it might be used as a biomarker for LC and widely promoted in clinical practice.

## Data Availability

Data is provided within the manuscript or supplementary information files. Find some help on our Data availability statements page.

## Abbreviations

GWAS: Genome wide Association Study
IVs: Instrumental variables
IVW: Inverse Variance Weighted
LC: Lung cancerLUAD lung adenocarcinoma
LUSC: Lung squamous cell carcinoma
MR: Mendelian randomization
NSCLC: Non-small cell lung cancer
SCLC: Small cell lung cancer
SNPs: Single nucleotide polymorphisms
UA: Uric acid
XOR: Xanthine oxidoreductase
